# Case report: Sublingual mucinosis in a dog

**DOI:** 10.3389/fvets.2022.986750

**Published:** 2022-10-26

**Authors:** Debora Tinto, Chiara Tassani, Matteo Di Benedetto, Silvia Sabattini, Ombretta Capitani

**Affiliations:** Department of Veterinary Sciences, University of Bologna, Bologna, Italy

**Keywords:** mucinosis, ranula, Shar-pei, sublingual swelling, ialuronic acid, case report

## Abstract

A 11-month-old male intact Shar-Pei (26. 5 kg) was presented for a bilateral sublingual swelling of 4 months duration. The exploration of the oral cavity highlighted the presence of bilateral sublingual swellings, primarily consistent with bilateral ranula. The bilateral disease was treated with two subsequent surgeries 4 weeks apart. During the surgery, after removing an elliptical portion of the mucosa of the sublingual swelling, the presence of gelatinous tissue was visualized, and no saliva was present. The result of histological exam was oral mucinosis. At the subsequent follow-up the dog was in excellent conditions, without any symptoms. 1 month after the last operation, the dog underwent a visit in sedation to better evaluate the oral cavity. Both surgical sites were well-healed and without the presence of relapses. Upon 8 months follow-up the patient remained free of disease. This is the first reported case of oral mucinosis in sublingual mucosa in dogs. In this case the surgical treatment was curative.

## Introduction

Oral focal mucinosis (OFM) is a rare clinic-pathological condition histologically similar to focal skin mucinosis and thus, OFM is considered as the oral counterpart of cutaneous focal mucinosis (1, 2).

Cutaneous mucinosis refers to an excessive deposition of mucinous substance in the dermis that clinically manifests as a thickening of the skin or as a vesicular appearance (3, 4).

A generalized cutaneous mucinosis, of likely genetic origin, occurs primarily in Shar-pei dogs, giving them its characteristic appearance (5). In other breeds, severe mucinosis is associated with secondary diseases such as intertrigo, bacterial infections and entropion, all of which can lead to severe deterioration in the health of the animal (6).

In human, OFM was first described and named by Tomich in 1974 (1). Its pathogenesis is still unclear, but an increased production of hyaluronic acid (HA) by fibroblasts in expense of collagen production and myxoid degeneration is suggested (7, 8).

Clinically, oral lesions often present as a small (< 1 cm) submucosal, single, pink, and slow-growing asymptomatic nodule containing gelatinous material (8, 9). The gingiva and palate are the most affected sites (9, 10).

OFM has already been reported in literature in a dog as a nodular lesion on the buccal mucosa (11). This is the first case described with this type of presentation.

## Case description

Written informed consent was obtained from the owner for the publication of this case report.

A 11-month-old male intact Shar-Pei (26.5 kg) was presented for a bilateral sublingual swelling of 4 months duration. The owners noticed that the was unable to masticate an pick up the food.

The patient had undergone bilateral entropion reduction surgery 3 months earlier. The dog was otherwise healthy with no known systemic diseases, and he was up to date on vaccines and flea and tick prevention. Two months prior to presentation, the dog was prescribed an anti-edema drug (Seaprose s) by the referring veterinary, without improvement.

At presentation, physical examination and vital parameters were within normal limits.

The exploration of the oral cavity with the patient under sedation highlighted the presence of bilateral sublingual swellings of soft and fluctuating consistency, primarily consistent with bilateral ranula.

Preoperative hematology and blood chemistry test were within normal limits. An ultrasound examination of the neck region was also performed to assess the integrity of the salivary glands. Chest x-rays were also performed. No remarkable alterations were observed.

**Figure 1:**
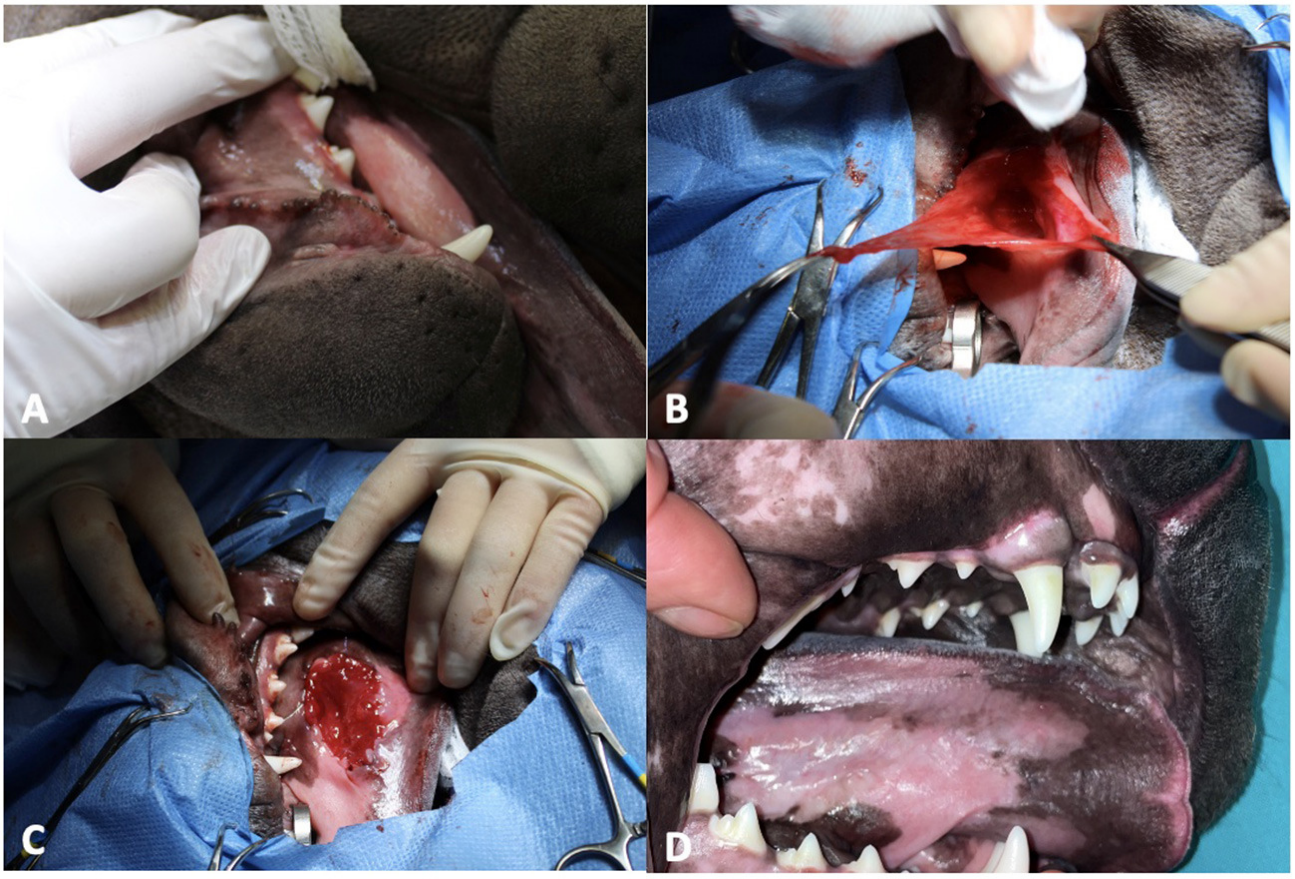
Right sublingual swelling at presentation. **(A)** Presence of gelatinous tissue visualized after removing portion of mucosa. **(B)** Marsupialization with absorbable monofilament suture 4–0 USP. **(C)** Complete healing 1 month after surgery. **(D)**.

During the surgery, with the patient in left lateral recumbency (Figure 1), after washing the oral cavity with diluted chlorhexidine, an elliptical portion of the mucosa of the right sublingual swelling was removed, the presence of gelatinous tissue was visualized, and no saliva was present. This tissue was partially removed, and the surgery ended with marsupialization in two simple continuous lines with absorbable monofilament suture 4–0 USP.

The removed mucosa and the gelatinous tissue were submitted for histological examination.

A post-operative therapy including antibiotic (amoxicillin and clavulanic acid, 20 mg/kg BID, for 7 days), NSAID (meloxicam, 0,1 mg/kg SID for 5 days) and anti-acid (omeprazole, 1 mg/kg SID for 7 days) was administered.

**Figure 2:**
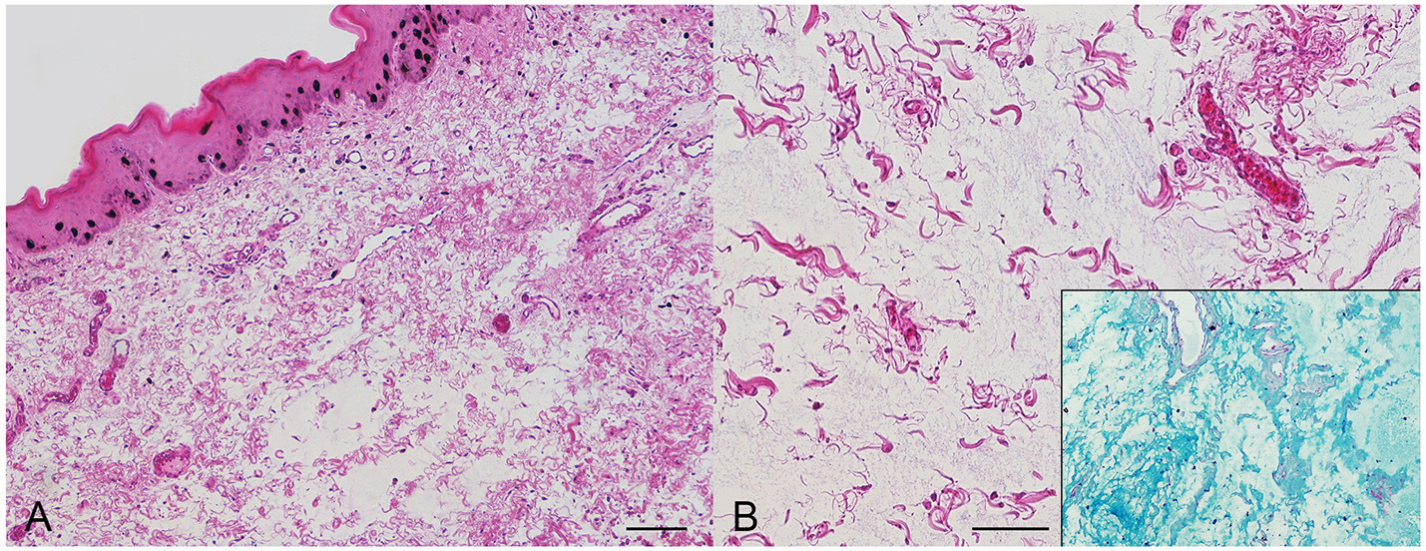
Dog, histologic preparation from a sublingual mass. **(A)** The subepithelial connective tissue is expanded by increased clear spaces. Hematoxylin and eosin. **(B)** At closer magnification, collagen bundles are separated by abundant, granular, amphophilic material, staining pale blue with alcian blue (inset), consistent with mucin. Hematoxylin and eosin. Bars, 100 μm.

Histologically, the subepithelial tissue was thickened and collagen bundles were distorted and widely separated by abundant, wispy, amphophilic material (mucin) and increased clear space (edema), admixed with granulation tissue, few lymphocytes and plasma cells. The overlying mucosal epithelium was mildly hyperplastic. The lacy material interpreted as mucin stained pale blue with alcian blue, whereas Periodic-acid Schiff reaction was negative, confirming its acid mucopolysaccharide composition (Figure 2).

Seven days later, at the follow-up visit, the dog showed no symptoms. The animal no longer exhibited difficulties in grasping and chewing food.

**Figure 3:**
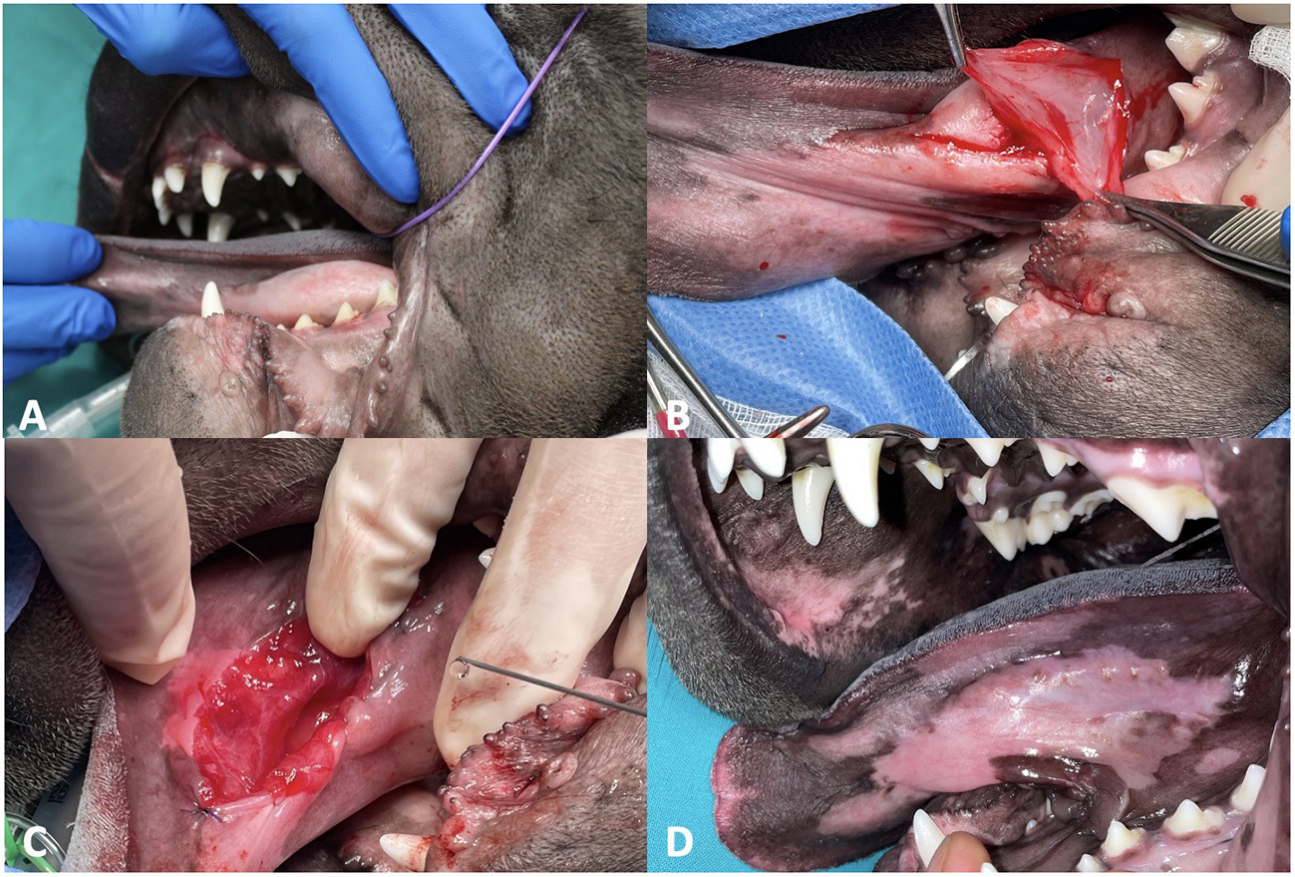
Left sublingual swelling at presentation. **(A)** Presence of gelatinous tissue visualized after removing portion of mucosa. **(B)** Marsupialization with absorbable monofilament suture 4–0 USP. **(C)** Complete healing 1 month after surgery. **(D)**.

One month after the first operation, the dog underwent surgical removal of the contralateral swelling with the same procedure (Figure 3). The site of the previous surgery was already completely healed and there was no sign of swelling.

In the light of the histological diagnosis, two skin biopsies with 4 mm Baker's punch were also performed.

The histological examination of the oral lesion confirmed the previous diagnosis. The examination of skin biopsies revealed a mild dermal thickening due to increased accumulation of pale-staining, fibrillar to granular mucin strands, thereby allowing to diagnose dermal mucinosis as well. At the subsequent follow-up the dog was in excellent condition, without any symptoms.

Under sedation oral examination performed 1 month after the last surgery revealed no evidence of relapse and surgical sites looked great. The patient remained disease free upon 8 months follow-up.

## Discussion

To our knowledge, only one case of OFM in dogs has been reported in literature. However, the lesion was a solitary asymptomatic nodule on the buccal mucosa (11). The dog was a 8-years old Labrador retriever with a single nodule on the buccal mucosa. It was round 1 cm diameter, raised and firm and an excisional biopsy was performed. A diagnosis of focal mucoid degeneratios was made and there has been no recurrence in the 12 months since surgery (11).

In human medicine the term OFM was first introduced in 1974 by Tomich, et al. (1) to describe lesions whose histopathological appearance was similar to the cutaneous counterpart. Since then, approximately 100 well-documented cases of OFM have been reported in the literature (12).

Studies have suggested that it is caused by the overproduction of HA by fibroblasts (1, 13). Although, the underlying cause for this overproduction remains largely unknown, a predisposing role of local trauma has been hypothesized in people (12, 13). The study of Cunha, et al. (12) failed to identify any apparent involvement of local irritation or masticatory trauma in most cases; however, three cases had local trauma involvement. These data support the conclusion that at least a subset of OFM may be reactive in nature. In our case, the traumatic cause cannot be completely excluded. However, it seems unlikely since owners did not report any trauma in the medical history and due to the bilateral presentation.

In human the most common intraoral sites of OFM are gingiva (58.2%), palate (15.3%), and alveolar ridge mucosa (8.2%), while in our case the lesion is found in the sublingual mucosa (12).

OFM manifests as a localized, sessile or pedunculated mucosal overgrowth, varying from few millimeters to 2 cm in size. They clinically mimic pyogenic granuloma, peripheral giant cell granuloma, peripheral ossifying fibroma, traumatic fibroma or focal gingival hyperplasia (14). We can also affirm that only the histological diagnosis is common to the human one, since the clinical presentation between man and dog, in this case, is completely different.

In our case, the lesion occurred as a bilateral sublingual swelling, similar in appearance to a ranula. While in the ranula the content is made up of saliva, in the current case there was the presence of gelatinous tissue, which was histologically and histochemically consistent with mucoprotein material rich in acid mucopolysaccharides. Before surgery it might have been advisable to carry out an FNA but the patient's age, anamnesis, localization, clinical symptoms and the findings of the oral visit convinced us to think of the presence of a bilateral ranula.

The surgery of sublingual mucocele entails the removal of the affected salivary gland in addition to marsupialitation. In this case, as ranula was the diagnosis at the time of the first surgery, we would have had to remove the salivary gland before marsupialization. But, after the ultrasound of the glandular complex of the neck showed no alterations in any salivary glands, at the behest of the owner, we only performed the marsupialization. Initially, the plan was to surgically repair both ranulas. However, upon visualization of gelatinous material instead of expected saliva-filled cavity, the decision of stage approach following histopathological evaluation was made.

Shar-pei dogs, more than other breeds, are predisposed to developing cutaneous mucinosis. In our case, skin biopsies were also performed, confirming the coexistence of both conditions.

It is presently unknown whether the oral and cutaneous localizations could be related, but being cutaneous disease a probably genetic based disease (15), this was strongly suspected.

A positive correlation is reported between increased HA serum concentrations and cutaneous mucinosis in dogs Zanna, et al. (15). In the present case, this test was not performed, but it would be interesting to assess whether increased serum HA levels can be associated with oral mucinosis as well.

In conclusion, in case of sublingual swelling in Shar-pei dogs or in dogs with suspected cutaneous mucinosis, oral mucinosis should also be considered as a differential diagnosis. In our case, the surgical treatment for this type of pathology was considered curative with a positive prognosis.

## Data availability statement

The original contributions presented in the study are included in the article/supplementary material, further inquiries can be directed to the corresponding author.

## Ethics statement

Ethical review and approval was not required for the animal study because the case report is a description of a clinical case. Written informed consent was obtained from the owners for the participation of their animals in this study.

## Author contributions

All authors listed have made a substantial, direct, and intellectual contribution to the work and approved it for publication.

## Conflict of interest

The authors declare that the research was conducted in the absence of any commercial or financial relationships that could be construed as a potential conflict of interest.

## Publisher's note

All claims expressed in this article are solely those of the authors and do not necessarily represent those of their affiliated organizations, or those of the publisher, the editors and the reviewers. Any product that may be evaluated in this article, or claim that may be made by its manufacturer, is not guaranteed or endorsed by the publisher.
